# Selfish Mitochondrial DNA Proliferates and Diversifies in Small, but not Large, Experimental Populations of *Caenorhabditis briggsae*

**DOI:** 10.1093/gbe/evv116

**Published:** 2015-06-24

**Authors:** Wendy S. Phillips, Anna L. Coleman-Hulbert, Emily S. Weiss, Dana K. Howe, Sita Ping, Riana I. Wernick, Suzanne Estes, Dee R. Denver

**Affiliations:** ^1^Department of Integrative Biology, Oregon State University; ^2^Department of Biology, Portland State University

**Keywords:** experimental evolution, genetic conflict, mitochondrial DNA, nematode, population size, selfish genetic elements

## Abstract

Evolutionary interactions across levels of biological organization contribute to a variety of fundamental processes including genome evolution, reproductive mode transitions, species diversification, and extinction. Evolutionary theory predicts that so-called “selfish” genetic elements will proliferate when the host effective population size (*N_e_*) is small, but direct tests of this prediction remain few. We analyzed the evolutionary dynamics of deletion-containing mitochondrial DNA (ΔmtDNA) molecules, previously characterized as selfish elements, in six different natural strains of the nematode *Caenorhabditis briggsae* allowed to undergo experimental evolution in a range of population sizes (*N* = 1, 10, 100, and 1,000) for a maximum of 50 generations. Mitochondrial DNA (mtDNA) was analyzed for replicate lineages at each five-generation time point. Ten different ΔmtDNA molecule types were observed and characterized across generations in the experimental populations. Consistent with predictions from evolutionary theory, lab lines evolved in small-population sizes (e.g., nematode *N* = 1) were more susceptible to accumulation of high levels of preexisting ΔmtDNA compared with those evolved in larger populations. New ΔmtDNA elements were observed to increase in frequency and persist across time points, but almost exclusively at small population sizes. In some cases, ΔmtDNA levels decreased across generations when population size was large (nematode *N* = 1,000). Different natural strains of *C. briggsae* varied in their susceptibilities to ΔmtDNA accumulation, owing in part to preexisting compensatory mtDNA alleles in some strains that prevent deletion formation. This analysis directly demonstrates that the evolutionary trajectories of ΔmtDNA elements depend upon the population-genetic environments and molecular-genetic features of their hosts.

## Introduction

Selfish genetic elements (SGEs) occur throughout the eukaryotic phylogeny and influence diverse evolutionary processes (Hurst and Werren 2001). Two features are required to identify DNA as “selfish.” First, the element must have a transmission advantage relative to the rest of the organism’s genome, often resulting in higher SGE abundances from one generation to the next than otherwise expected. Second, the element must be either neutral or deleterious to the organism; it cannot provide a fitness advantage. SGE examples include transposons, supernumerary nuclear chromosomes, meiotic driver genes, and the genomes of intracellular bacteria and organelles (Hurst and Werren 2001). Some SGEs cause severe sex ratio distortion (Randerson et al. 2000; Tao, Araripe, et al. 2007; Tao, Masly, et al. 2007; Larracuente and Presgraves 2012) and are hypothesized to underlie speciation processes through hybrid dysfunction (Presgraves 2010). Empirical results from analyses of closely related *Drosophila* species infected with *Wolbachia* support this hypothesis (Cordaux et al. 2011; Veneti et al. 2012), as do other studies implicating organellar cytoplasmic male sterility factors in the speciation of plants (Rieseberg and Blackman 2010; Fujii et al. 2011). SGEs can impact the expression of nearby genes (Antonaki et al. 2011; Rebollo et al. 2011) and the overall evolution of genome architecture (Lynch and Conery 2003).

Organelle genomes generally undergo uniparental inheritance (through the female gamete) in most eukaryotic species studied. Although maternal inheritance is thought to facilitate coevolution of organelle and nuclear genomes, it also renders cytoplasmic DNA susceptible to Muller’s ratchet dynamics and associated accumulation of deleterious variants, including SGEs (Aanen et al. 2014). Organelle-housed SGEs are most frequently discovered and studied in fungal and plant species. In plants, some mitochondrial SGEs lead to cytoplasmic male sterility phenotypes (Rieseberg and Blackman 2010; Fujii et al. 2011). Selfish evolutionary behavior of mtDNA might be rare in metazoans because most animals have separate sexes (Aanen et al. 2014), and as a consequence of the small sizes and streamlined contents of most animal mitochondrial genomes (Galtier 2011). However, if widely masked by fixed nuclear restorer loci (Dowling et al. 2007; Luo et al. 2013), cryptic mitochondrial SGEs might be more prevalent in animals than is currently thought.

What factors influence the evolutionary success (or demise) of cytoplasmic SGEs? This question has received theoretical attention (Cosmides and Tooby 1981; Otto and Hastings 1998) and a simulation analysis demonstrated that the accumulation of SGEs increases as a function of decreasing host population size (Rispe and Moran 2000). An empirical *Saccharomyces cerevisiae* experimental evolution study demonstrated that levels of selfish mutant mitochondrial DNA (mtDNA) molecules, with associated respiration defects, remained high only when the yeast population size was small (Taylor et al. 2002) and selection at the level of mtDNA overpowered selection at the level of the individual. At larger experimental population sizes, strong selection at the level of the individual, favoring yeast cells with effective respiration, resulted in lower levels of selfish mtDNA. This lab mutant study provided the first empirical evidence that cytoplasmic SGEs proliferate under small host population size and decline when population size is large. Many questions remain, however, regarding the evolution of SGEs. How frequently and under what evolutionary conditions do new SGEs arise? Do different populations within a species vary in their susceptibilities to SGEs?

**Fig. 1.**
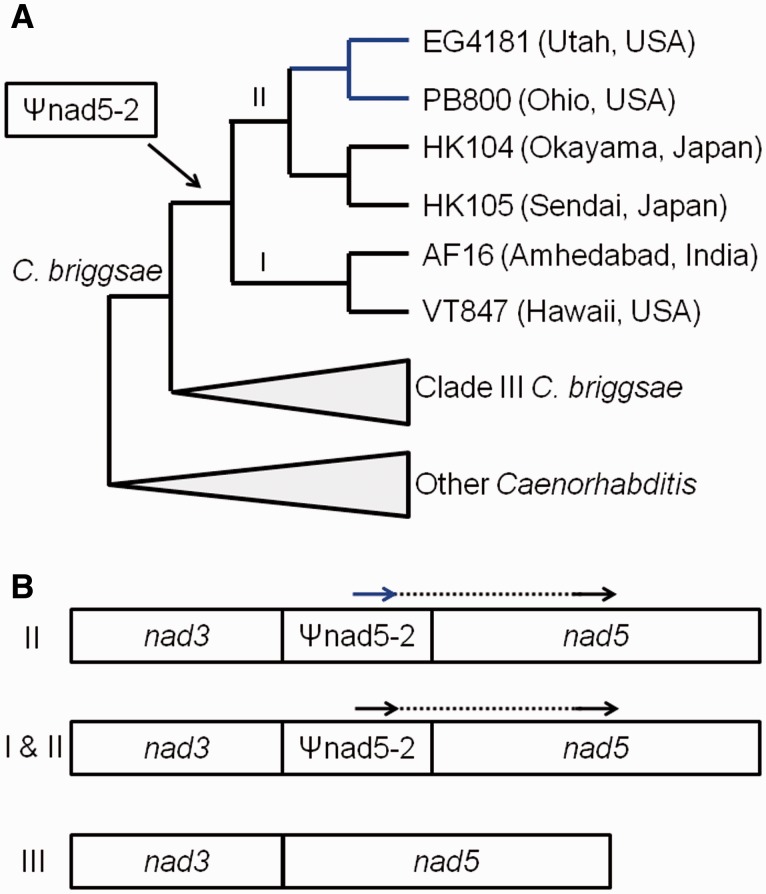
— Phylogenetic relationships based on mtDNA of the six *C. briggsae* natural strains used for experimental evolution progenitors (*A*) and schematic of the ΔmtDNA-C locus (*B*). In (*A*), Roman numerals indicate the intraspecific mtDNA clade designations defined in Raboin et al. (2010). Clade I is also commonly referred to as the “tropical” clade, Clade II as the temperate clade, and Clade III as the “equatorial” Clade (Cutter et al. 2006). The blue branches lead to strains that encode the putative compensatory mutations associated with the ΔmtDNA-C locus (Howe and Denver 2008). In (*B*), schematics of the *nad5* deletion region are shown; Roman numerals on the left indicate the intraspecific clades (sensu Raboin et al. 2010) in which different states are observed. The arrows indicate positions of the 21-bp direct repeats associated with deletion formation. The dashed line indicates DNA sequences missing in ΔmtDNA-C molecules. The blue arrow indicates the position of the direct repeat bearing putative compensatory mutations (observed in some Clade II strains). Clade III strains lack Ψnad5-2 elements and associated deletions, as indicated in the bottom mtDNA gene model. All Clade I strains and some Clade II strains have perfect direct repeats.

*Caenorhabditis briggsae* nematodes offer an effective system for investigating diverse evolutionary questions including reproductive mode evolution (Dolgin et al. 2007; Woodruff et al. 2010; Guo et al. 2013; Chen et al. 2014), speciation (Baird 2002; Dolgin et al. 2007; Woodruff et al. 2010; Baird and Stonesifer 2012; Kozlowska et al. 2012), and within-organism genetic conflict (LaMunyon and Ward 1997; Clark et al. 2012). We discovered the natural occurrence of mtDNA molecules containing a large deletion (ΔmtDNA), characterized as a cytoplasmic SGE (Howe and Denver 2008; Clark et al. 2012), in *C. briggsae* natural populations. Here, we refer to this particular deletion as the canonical ΔmtDNA (ΔmtDNA-C) because new ΔmtDNA types will be reported later in the Results. Our initial study (Howe and Denver 2008) revealed that most, but not all, of the then-known *C. briggsae* natural strains harbored heteroplasmic mixtures of intact “wild-type” mitochondrial genomes and ΔmtDNA-C molecules. ΔmtDNA-C is missing 871–887 bp (depending on the *C. briggsae* strain) of sequence, including part of a pseudogene element named ψnad5-2 (Raboin et al. 2010) as well as highly conserved nucleotides in the *nad5* protein-coding gene (fig. 1). Further analyses revealed that ΔmtDNA-C molecules increase in frequency and different ΔmtDNA molecule types arose at high rates in *C. briggsae* lines bottlenecked in the lab (Howe et al. 2010; Clark et al. 2012).

A 21 bp direct repeat flanks the sequence deleted by ΔmtDNA-C, with one copy in ψnad5-2 and a second downstream in *nad5* in intact mtDNA. With the formation of the ΔmtDNA-C deletion, only one of those repeat sequences remains (fig. 1). Humans and other animals accumulate similar direct repeat-associated ΔmtDNA in somatic cells as they age (Oliveira et al. 2010). This pattern (flanking direct repeats) is consistent with illegitimate recombination as the mechanism of ΔmtDNA-C formation, as previously invoked in plant-parasitic nematode species where mtDNA occurs as numerous minicircles (Armstrong et al. 2007; Gibson, Blok, Dowton, et al. 2007; Gibson, Blok, Phillips, et al. 2007; Hoolahan et al. 2012). Imperfect ψnad5-2 repeat units (with two SNPs relative to the unit present downstream in *nad5*) occur in some *C. briggsae* strains from North America and Europe that are part of the “temperate” intraspecific clade (Clade II in fig.1). These sequences constitute putative compensatory alleles because strains with these mtDNA SNPs harbor significantly lower levels of ΔmtDNA-C as compared with those with perfect 21 bp repeats (Howe and Denver 2008). The occurrence of such alleles in some, but not all strains leads to the expectation that different *C. briggsae* lineages will vary in their susceptibility to ΔmtDNA accumulation.

The *C. briggsae* ΔmtDNA-C elements constitute a rare known case of animal mitochondrial SGEs. These circles experience an approximately 1% per-generation transmission advantage relative to the larger intact mtDNA molecules that coexist in mitochondria with them (Clark et al. 2012). Some *C. briggsae* strains harboring higher ΔmtDNA-C levels were found to have significantly reduced fecundity and pharyngeal pumping rates compared with those with lower levels (Estes et al. 2011). In vivo subcellular assays showed that the strain with the highest ΔmtDNA-C level, HK105, suffered greater levels of endogenous reactive oxygen species (ROS); lab-generated mitonuclear hybrid strains (“cybrids”) demonstrated that mtDNA was a major driver of the elevated ROS (Hicks et al. 2012). However, VT847, another strain harboring high ΔmtDNA-C levels, had fecundity more typical of other *C. briggsae* natural strains and normal ROS levels (Estes et al. 2011; Hicks et al. 2012). There is no evidence, however, that ΔmtDNA-C confers any benefit to the nematodes in lab studies. Thus, these lab-based studies provide evidence that *C. briggsae* ΔmtDNA-C elements satisfy both the transmission advantage and deleterious or neutral fitness effects requirements necessary to characterize DNA as selfish. This naturally occurring selfish mtDNA system has the potential to provide insights into the evolutionary parameters that govern the evolution of cytoplasmic SGEs in metazoans.

**Fig. 2.**
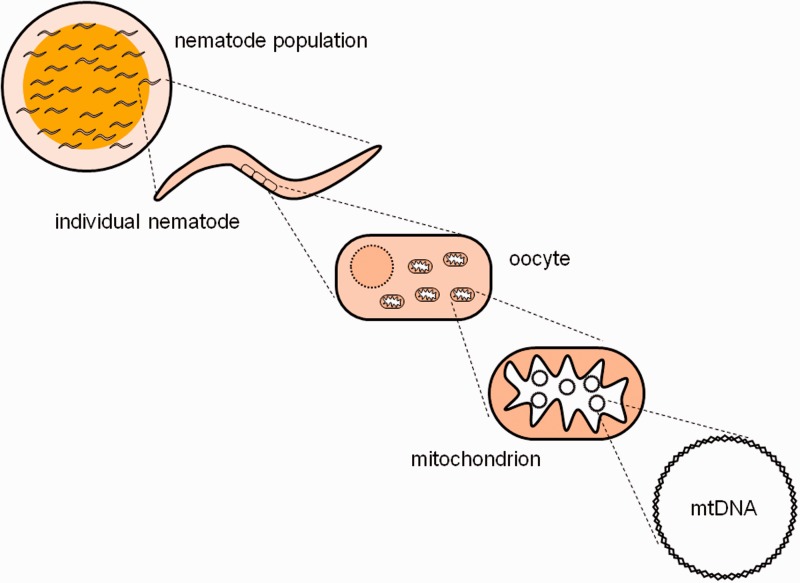
— Levels of evolution schematic. The diagram illustrates hierarchical biological levels of organization in the study system, from laboratory *C. briggsae* populations to mtDNA molecules.

Here, we investigate the transgenerational dynamics of *C. briggsae* ΔmtDNA molecules using an experimental evolution approach. Figure 2 provides an overview of the system under study and the associated levels of evolution, from laboratory nematode population to mtDNA molecule. Our experiments included four different experimental population sizes and six different *C. briggsae* natural-strain genotypes. We used polymerase chain reaction (PCR) and DNA sequencing analyses to identify and characterize the state of mtDNA across multiple generational time points. This approach identified many new ΔmtDNA variants, some originating during the course of the evolution experiment, and provided new insights into the evolution of cytoplasmic SGEs in different host genetic backgrounds and at varying host population sizes.

## Materials and Methods

### Experimental Evolution

Five replicate populations of six different natural *C. briggsae* strains (table 1) underwent independent laboratory evolution for 50 generations or until extinction in an array of population (bottleneck) sizes: *N* = 1, 10, 100, and 1,000, for a total of 120 evolving lineages. All nematodes were cultured at 25°C ± 1°C on standard nematode growth media with 200 mg streptomycin sulfate per liter and fed *Escherichia coli* strain HB101. At five-generation intervals, nematodes of mixed ages were collected from each replicate into 20 μl lysis buffer and stored at −80°C for later molecular genetic analysis. For the *N* = 1 and *N* = 10 lineages, nematodes were expanded for one or two generations prior to harvesting in order to obtain sufficient biological material for molecular analysis.

**Table 1:** *Caenorhabditis briggsae* Strains Used for Experimental Evolution

Strain	Origin	Clade	CM
AF16	India	I (Tropical)	−
EG4181	Utah	II (Temperate)	+
HK104	Japan	II (Temperate)	−
HK105	Japan	II (Temperate)	−
PB800	Ohio	II (Temperate)	+
VT847	Hawaii	I (Tropical)	−

Note.—Clade indicates the intraspecific mtDNA clade sensu Raboin et al. (2010). CM indicates the presence (+) or absence (−) of a putative compensatory mutation expected to reduce the likelihood of ΔmtDNA-C formation.

Replicate populations of *N* = 1 and *N* = 10 were initiated from a single randomly selected hermaphrodite whose remaining offspring were cryogenically preserved as generation zero (G0). Replicates were maintained by transferring either one or ten L4-stage hermaphrodites to a new 35 mm Petri plate every 48 ± 2 h. If a hermaphrodite in the *N = 1* treatment laid no eggs within this timeframe, an additional 2 days was allowed for reproduction. If it failed to reproduce after this time, another of its kin was selected for propagation (considered “going to backup,” GTB). A line was considered extinct after five consecutive failures of the GTB procedure.

Populations of *N* = 100 and *N* = 1,000 were propagated by harvesting all eggs from each replicate and transferring the desired number to a new plate every 48 ± 2 h. Populations were cultured on 60 and 100 mm Petri plates, respectively. Specifically, nematodes from replicates were collected into 5 ml M9 buffer and 2 ml of 2:1 (v:v) 6% bleach:5 M NaOH solution was added to dissolve the cuticles. Eggs were rinsed twice and resuspended in M9 buffer. Pellets of eggs were transferred with 1 ml fresh M9 to a 1.7 ml microcentrifuge tube to achieve uniform dispersion within a column. After vortexing briefly, egg density was calculated for each replicate from eight 1 ul samples and the volume appropriate to achieve the desired population bottleneck size was transferred to a fresh Petri plate. Accuracy of calculated egg densities was determined by counting the surviving worms of one replicate per strain immediately prior to transfer.

### Male Frequency Analysis

After observing an increased prevalence of males within the AF16 lines evolving at *N* = 1,000, we followed the approach of a previous study (Denver et al. 2010) to estimate male frequency at G41 in the *N* = 1,000 evolving lines of all six strains. Male frequency was scored by scoring sex for 150 young adult individuals from five plates per line (a total of 150 plates). Plates were prepared by transferring a volume of eggs expected to yield approximately 200 individuals.

### Molecular Analysis of mtDNA

DNA was extracted from pools of approximately 50–500 worms (with a few exceptions when only approximately 10 worms were available) using a standard proteinase K-based lysis buffer method. In order to detect mtDNA size variants, the whole mtDNA genome was amplified via four overlapping PCR fragments using previously described primers (37 F-36 R, 11 F-72 R, CbMt_13F- CbMt_6R, and 39 F-4 R; Howe and Denver 2008; Howe et al. 2010) and MyTaq proofreading DNA Polymerase (Bioline). The approximately 2,800–4,900 bp products were analyzed by electrophoresis on 1% agarose gels (fig. 4*A*). The presence of any bands which differed in expected size from wild-type or ΔmtDNA-C were recorded. To determine the location of deletions, PCR products smaller than intact mtDNA from at least one generation of each line in which they appeared were sequenced using BigDye (Applied Biosystems) with standard reaction conditions. If only a band of the smaller, deletion-bearing mtDNA was produced, that PCR product was purified using magnetic beads before sequencing. When PCR amplification produced amplicons from both intact mtDNA and deletion containing mtDNA, the smaller, deletion-bearing band was isolated using a QIAquick gel extraction kit in preparation for sequencing. In cases where sequencing from gel extracted products failed, the PCR products were cloned into the pCR8/GW/TOPO vector (Life Technologies). The resulting clones were screened to find those with plasmids of the predicted size for containing the deletion PCR product. Plasmid DNA was purified with the QIAprep spin miniprep kit (Qiagen) for sequencing. To determine deletion boundaries, sequences were aligned with the AF16 reference mtDNA sequence in GenBank (AC186293) if the deletion occurred in AF16 or with sequences from Howe and Denver (2008) from the strain in which they occurred. After long PCR identification of deletion-containing molecules, follow-up PCRs were performed to more specifically identify and characterize the ΔmtDNA.

Most newly detected deletions were in the same mitogenomic region as ΔmtDNA-C (table 2), but when the deleted sequences included the 58R priming site used for our previously developed ΔmtDNA-C assays (see below), their amplification was blocked in the Cb_Mt1F and 58 R PCR screen. To determine the generation at which these deletions could first be detected, new primers were designed to amplify both ΔmtDNA-C (yielding a approximately 540 bp product) and the new deletions in a single reaction but with differently sized amplicons (163–643 bp) representing the new ΔmtDNA type. Due to between-strain differences, two primer sets were designed within a few base pair from each other: Cb_Mt102F 5′-AGAAGGTGGTAGCCTTGAG-3′ and Cb_Mt105R 5′-TAAAGAAATAATCTAGGTACATTGAGT-3′ designed for strains EG4181, HK104, HK105, and PB8000; and Cb_Mt103F 5′-GGTAACCTTGAGGTCACTG-3′and Cb_Mt106R 5′-ATAAAGAAATAATCTAGGTACATTAAG-3′ designed for strains AF16 and VT847. For those lines in which a new deletion could be amplified by these primers (ΔmtDNA1-6), all generations were subjected to PCR to determine the first generation in which the deletion could be detected. PCR products were electrophoresed through 2% agarose gels, stained with ethidium bromide, and photographed for manual scoring. The consistency in the results of our analysis suggests that PCR artifacts are not a significant concern. This is evidenced by the uniformity of results between independent initial long PCRs and follow-up standard PCRs, and the cross-generational patterns of ΔmtDNA persistence (see Results). New DNA sequences were submitted to GenBank under accession numbers KR185945–KR185961.

**Table 2:** ΔmtDNA Variants Detected

Var.	Size (bp)	Loci	Strain(s)	Pop. Size	No. Occ.	Repeat
C	869	Ψnad5–2, *nad5*	All Six			AGGGTTTCAATAGTTACTTT
1	540	*nad5*	HK105	10	1	N/A
2	765	*nad5*	HK104	1	1	GATTATTTC
3	870	Ψnad5–2, *nad5*	AF16	1	1	CCTTGAGG
4	976	*nad5*	HK105	10	1	ATTTTTT
5	1,135	*nad5*	Five^a^	1, 10	8	GGATTTTT
6	1,137	*nad5*	HK104, VT847	10	2	GATTAAA(A/T)TTTAATTT
7	1,212	*nad5*	HK104	1	2	ATTGGATT
8	1,262	Ψnad5‐2, *nad5*	HK104	1	2	TTATTTTT
9	1,446	*cytb*, *trnL*, *trnT*, *nad4*	HK105	100	1	TAAGACTA

Note.—Var. provides a designator to accompany each specific ΔmtDNA variant. Size is the length of the deletion in basepairs. Loci indicates the mtDNA genes and other elements affected by the deletion. Strain(s) refers to the specific *C. briggsae* isolates in which different variants were observed. Pop. Size shows the population sizes in which the variants were respectively detected. No. Occ. indicates the numbers of independent experimental lineages in which ΔmtDNA variants were observed. Repeat shows the direct repeat DNA sequence motifs associated with different ΔmtDNAs.

**Fig. 3.**
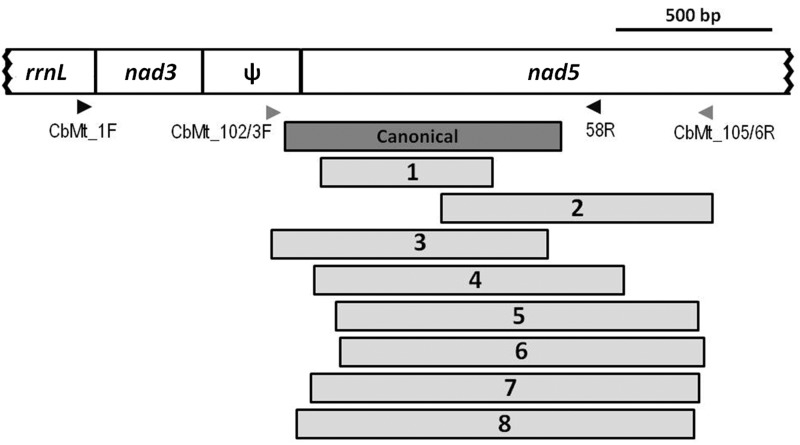
— Locations of newly detected ΔmtDNA molecules. The numbers in the rectangles correspond with the numbers assigned to the deletions in table 1. Genes are named in the diagram and ψ indicates the ψnad5-2 element. One variant, ΔmtDNA-9, is not shown because its deletion boundaries are on the opposite side of the mtDNA chromosome (table 2). The arrowheads show the positions of primers used; black arrowheads indicate original primer set for analyzing ΔmtDNA-C, CbMt_01F-58 R (see Materials and Methods). The gray arrowheads indicate the new primers (102 F, 103 F, 105 R, and 106 R) were designed to discriminate between ΔmtDNA-C and new ΔmtDNA types.

^a^ΔmtDNA-5 was observed in all strains except EG4181. Mitochondrial genome locations of ΔmtDNA elements are shown in figure 3.

To assay levels of ΔmtDNA-C, we employed a PCR analysis designed to determine proportions of deletion-bearing mtDNA to intact mtDNA previously described by Howe and Denver (2008) and Clark et al. (2012). Briefly, a standard PCR reaction was performed with primers Cb_Mt1F and 58 R, which span the deletion and result in one of three banding patterns: an “intact” approximately 1,700 bp wild-type band, a “deletion” approximately 800 bp ΔmtDNA-C band, or an “intermediate” pattern with both wild-type and ΔmtDNA-C bands. Reactions resulting in a single detectable band do not necessarily indicate a homoplasmic template because of complicated amplification dynamics when samples contain mixed templates of varying proportions that yield differently sized amplicons. PCR products were electrophoresed on standard 1% agarose gels, stained with ethidium bromide, and photographed for manual scoring. We note that the previous correlational analyses relating standard PCR and qPCR methods for estimating ΔmtDNA-C levels (Howe and Denver 2008) were based on DNA samples from individual L1-stage nematodes whereas our current assays used genomic DNA samples prepared from many mixed-stage nematodes. Thus, the specific quantitative associations reported in Howe and Denver (2008) might not directly extend to this study.

We also provided a high-throughput DNA sequencing-based analysis of ΔmtDNA occurrence (supplementary fig. S1, Supplementary Material online), involving strain AF16 and a derivative line evolved at *N* = 1 for 50 generations, for the purpose of further corroborating the presence of ΔmtDNA in this species using a complimentary experimental method. The specific bottlenecked AF16 line was from a different lab evolution experiment relative to those that were the focus of this paper. For this analysis, thousands of L1-stage nematodes were collected subsequent to standard hypochlorite treatment of large mixed-stage populations (kills all animals except embryos) for each of AF16 (unevolved) and its derivative bottlenecked line. DNA was extracted using a Qiagen DNeasy kit. This total DNA was then analyzed on an Illumina MiSeq system available at the Oregon State University Center for Genome Research and Biocomputing. Sequencing was carried out as paired-end 150 bp reads. After sequencing, reads were mapped to the reference *C. briggsae* AF16 genome sequence (Stein et al. 2003) using CLC Genomics Workbench software (www.clcbio.com, last accessed July 1, 2015) and under strict mapping parameters (98% length identity and 98% sequence identity). The Illumina data were archived with the NCBI Short Read Archive under accession numbers SRX1014084 and SRX1014093.

### Statistical Analyses of ΔmtDNA Evolution

**Fig. 4.**
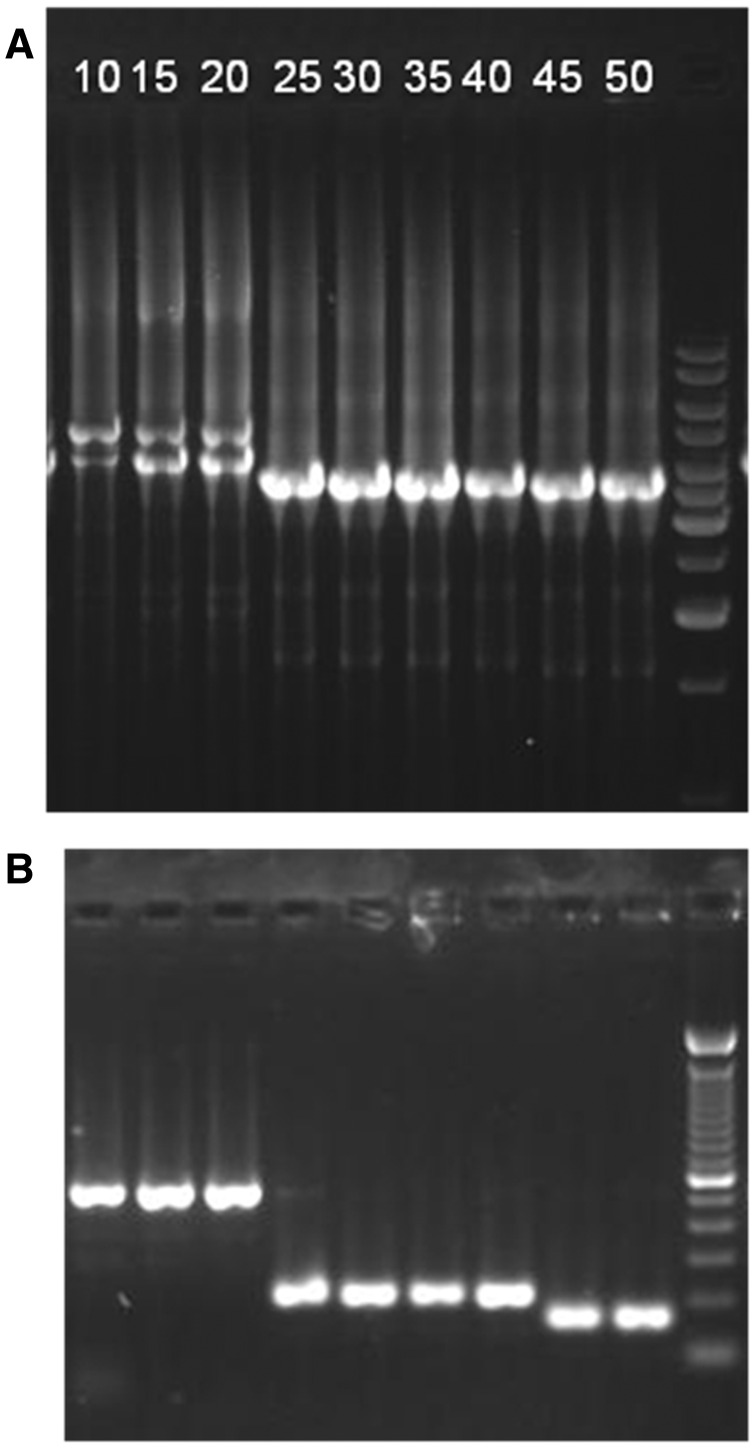
— Detection and characterization of ΔmtDNA. Electrophoresis analysis of mtDNA length variation in generations 10–50 (lanes 1–9, white numbers across the lanes label the generation) for a *N* = 1 sample from *C. briggsae* strain HK104. CM+ indicates a strain with a putative compensatory mutation in the Ψnad5-2 element (see fig. 1); CM− indicates a strain that lacks a compensatory mutation in Ψnad5-2. The 1 kb+ molecular marker (Invitrogen) is shown in the far right lane of (*A*), the 100 bp molecular marker (Invitrogen) is shown in the far right lane of (*B*). Long-PCR amplification results are shown in (*A*), displaying the heteroplasmic co-occurrence of a wild-type mtDNA molecules (larger amplicon) along with ΔmtDNA-C (smaller amplicon), in early generations (lanes 1–3). The detection of a PCR product associated with a new smaller mtDNA deletion (ΔmtDNA-7) can be seen at generation 25. A more subtle size shift toward a new even smaller ΔmtDNA (ΔmtDNA-8) occurs between generations 40 and 45. Follow-up standard PCR amplification of the same samples is shown in (*B*) using a set of primers closer to the focal deletion region. The canonical deletion produces a 540 bp band, ΔmtDNA-7 a 197 bp band, and ΔmtDNA-8 a 163 bp band.

**Fig. 5.**
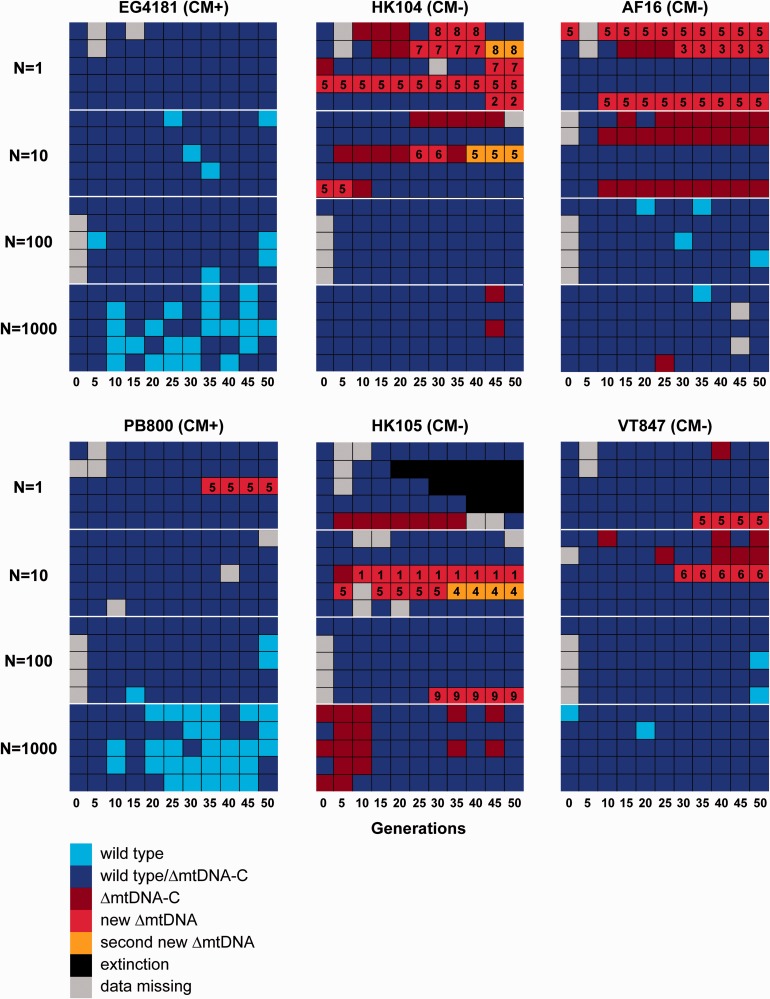
— Composition of mtDNA at five-generation intervals within experimental lines across 6 strains, 50 generations, and 4 population sizes. Each square shows mtDNA composition within a single experimental line measured at a particular five-generation interval. Light blue squares indicate that only wild-type mtDNA genomes were detected, dark blue squares indicate that a mixture of wild-type/ΔmtDNA-C was detected, and dark red squares indicate that only ΔmtDNA-C PCR banding types were detected. A transition from dark blue to light blue indicates a decrease in ΔmtDNA-C between time points; a transition from dark blue to dark red indicates an increase in ΔmtDNA-C. Appearance of new or previously undetectable deletions is represented by bright red squares; the number within these squares corresponds to those in figure 3 and table 2, describing each of these deletions. Orange squares denote the detection of a second new or previously undetected deletion within a line. Black squares indicate line extinctions. Gray squares indicate missing data.

An ordered logistic regression model (Agresti 2002) was used to analyze the deletion score data. The PCR banding patterns were scored 1–5. Scores of 1–3 reflected ΔmtDNA-C gel banding assay results (1 = wild-type PCR product only, 2 = both wild-type and deletion products, 3 = deletion product only) following Clark et al. (2012). A score of 4 indicated the detection of a new ΔmtDNA type (ΔmtDNA 1-9 in table 2). A score of 5 was assigned when a second distinct ΔmtDNA type appeared and supplanted evidence of a different new ΔmtDNA type observed in a previous generation (figs. 4*B* and 5). The analysis was implemented with the R package “ordinal” (version 2012.09-11) using the “clmm2” function, and maximum likelihood estimates of the parameters were approximated using the adaptive Gauss–Hermite quadrature method with 20 quadrature nodes. Replicate line was modeled as a random effect and assumed to be independent and normally distributed. Likelihood ratio tests were used to select the most parsimonious model by singly dropping each predictor from the full model and then testing significance of possible pairwise interactions among all three predictors (“population size” and “strain,” categorical predictors; “generation number,” continuous predictor; supplementary table S2, Supplementary Material online). Odds ratios reported in table 3 were calculated by exponentiating the predictor coefficients estimated by the model, with strain EG4181, *N* = 1,000 used as the reference group. All observations from HK105 were excluded from this analysis due to high prevalence of missing data at population size of 1.

**Table 3:** Results of Statistical Analysis

Variable	Odds Ratio (95% CI)	Model Estimate	SE	Wald *z*	*P* Value
Pop size = 1	21.97 (2.71–178.2)	3.090	1.068	2.894	0.004
Pop size = 10	27.36 (3.65–204.9)	3.309	1.027	3.222	0.001
Pop size = 100	3.95 (0.46–33.63)	1.373	1.093	1.256	0.209
Strain = PB800	2.70 (0.46–15.68)	0.993	0.898	1.106	0.269
Strain = VT847	46.57 (6.94–312.81)	3.841	0.971	3.955	< 0.001
Strain = AF16	232.06 (31.44–1712.24)	5.447	1.020	5.341	<0.001
Strain = HK104	1084.64 (108.75–10827.08)	6.989	1.174	5.955	<0.001
Generation	0.96 (0.94–0.99)	−0.039	0.013	−3.044	0.002
Gen x (Pop size = 1)	1.16 (1.11–1.22)	0.151	0.023	6.578	<0.001
Gen x (Pop size = 10)	1.08 (1.05–1.12)	0.082	0.018	4.481	<0.001
Gen x (Pop size =100)	0.98 (0.94–1.03)	−0.020	0.024	−0.804	0.421

Note.—Maximum likelihood estimates from fit of ordered logistic regression mixed model. Strain = EG4181 and Pop size = 1,000 are the reference categories for the categorical predictors. HK105 was excluded because extinctions caused extensive missing data at *N* = 1. *P* values are based on the Wald statistic.

## Results

### Experimental Evolution

We propagated 120 sets of laboratory nematode populations for this study involving four experimental population sizes (*N* = 1, 10, 100, and 1,000) and six *C. briggsae* natural strains (fig. 1 and table 1), all known to harbor ΔmtDNA-C at different levels. Our earlier work (Howe and Denver 2008) showed that the two strains (EG4181 and PB800) encoding putative compensatory mtDNA alleles harbored lower ΔmtDNA-C levels (0–5% of total mtDNA) relative to the other four strains. The highest ΔmtDNA-C levels were observed in HK105 and VT847 (40–50% of total mtDNA); intermediate levels (5–20% of total mtDNA) were observed in AF16 and HK104. Five biological replicate populations were maintained for each strain x population size combination examined in this study. Each lab population evolved for a maximum of 50 generations and nematode samples were collected every five generations for mtDNA analysis. Laboratory procedures for experimental evolution are described in the Materials and Methods.

We analyzed among-strain variation in extinction, successful transfer frequencies, developmental delay, and mortality for the nematodes evolved at *N* = 1. Lineage extinction prior to the maximum target generation (G_max_ = 50) was observed for only one *C. briggsae* strain (HK105), in three-fifth replicate lines of this strain. Extinction in these three cases occurred at 14, 25, and 36 generations, respectively. All replicates of all strains at the three larger population sizes (*N* = 10, 100, and 1,000) achieved G_max_ without extinction. The frequency of transfers successfully establishing new generations for each replicate was quantified as the ratio of the number of transfers to G_max_ (see Materials and Methods), following (Joyner-Matos et al. 2011). This ratio was lowest among experimental lines of HK105 (0.83), followed by HK104 (0.89), AF16 (0.97), PB800 (0.98), EG4181 (0.99), and VT847 (1.0). HK105 experimental lines also exhibited a reproductive delay; the time between transfers (i.e., generations) was longer for HK105 (3.51 ± 0.59 days) than for all other strains (2.10 ± 0.04 days) in the *N* = 1 treatment. Finally, we recorded whether transferred hermaphrodites survived until the following transfer (when one of their offspring was selected for transfer). The resulting mortality patterns mirrored those of failed transfers reported above. HK105 performed worst with 16% mortality per transfer (one-quarter of which resulted from premature hatching of eggs within the parent). Replicates of HK104 showed 7% mortality, followed by AF16 (3%), EG4181 (0.004%), and PB800 and VT847 (both 0%).

During lab evolution, we noted an increased presence of males within replicate lineages of AF16 evolving at *N* = 100 and 1,000 as early as G3 and G14, respectively. An analysis of male frequency conducted at G41 for all *N* = 1,000 lineages revealed average male frequency in AF16 lines to be 22.1% as compared with approximately 0% in all other strains (supplementary table S1, Supplementary Material online). These AF16 males were in several instances observed to be copulating with hermaphrodites. Thus, outcrossing may have been a significant contributor to evolution in the AF16 lineages but not to that in the other strains. Furthermore, unlike the *N* = 1 and 10 treatments where hermaphrodites were directly transferred each generation, the *N* = 100 and 1,000 lines were maintained by bulk population transfers (see Materials and Methods) that may have included male offspring. Our observations and analysis suggest that this was a rare occurrence for strains other than AF16. To the extent that male offspring were included in the AF16 population transfers, the actual *N_e_* of AF16 lineages could differ compared with other strains.

### Mitogenome-Wide Screen for ΔmtDNA

Our previous studies (Howe and Denver 2008; Howe et al. 2010; Clark et al. 2012), which used a variety of conventional PCR and quantitative real-time PCR approaches, provided many points of evidence that ΔmtDNA-C is an SGE located in mitochondria, and against the possibility that ΔmtDNA-C is a nuclear-mitochondrial pseudogene. For this study, we performed a high-throughput DNA sequencing analysis to provide an additional experimental view and confirmation of ΔmtDNA-C. Nematodes at the L1 larval stage were analyzed for the ancestral AF16 strain and for one AF16 line bottlenecked at *N* = 1 for 50 generations, using Illumina MiSeq technology (see Materials and Methods for details). Read coverage at the *nad5* sequences deleted in ΔmtDNA-C molecules were much lower in the bottlenecked line compared with that in the ancestral line (supplementary fig. S1, Supplementary Material online). This pattern is consistent with the presence of ΔmtDNA-C molecules in *C. briggsae*, as previously reported using PCR methodologies (Howe and Denver 2008; Clark et al. 2012).

We next assayed for ΔmtDNA across all five-generation time points (where possible) in all experimental populations of *C. briggsae.* The whole approximately 14.4 kb mitochondrial genome was long PCR-amplified as four overlapping amplicons, ranging in size from 2.8 to 4.9 kb (see Materials and Methods). When size variants were detected on agarose gels, amplicon sequencing was conducted to reveal the DNA sequence characteristics of the variant. New PCR primers were developed as needed to more specifically characterize newly discovered mtDNA variants. This approach was used to comprehensively search for new ΔmtDNA molecules across all mtDNA regions. We also implemented a conventional PCR assay developed (Howe and Denver 2008; Clark et al. 2012) to coarsely characterize the relative heteroplasmic frequencies of ΔmtDNA-C molecules (but that cannot accurately evaluate levels of other new ΔmtDNAs observed here).

New ΔmtDNA variants (containing deletion boundaries that differed from ΔmtDNA-C) were detected in 19 different experimental *C. briggsae* lineages. Nine different specific ΔmtDNA sequence types were identified among these 19 lab lineages (table 2), with deletions ranging in size from 539 to 1,445 bp. Like ΔmtDNA-C, the majority of the newly identified ΔmtDNA sequence types (8/9) were detected in the *nad5* gene region (fig. 3). Similar to previous observations in a set of long-term bottlenecked *C. briggsae* lines (Howe et al. 2010), in this study some ΔmtDNA types were detected in multiple independently evolved lines of the same strain, suggesting that these probably constitute preexisting heteroplasmic variants present in the progenitor that experienced differential segregation patterns among lab lines. New ΔmtDNA types were found in eleven experimental lines in population size *N* = 1, seven in *N* = 10, one in *N* = 100, and zero in *N* = 1,000.

After initial detection of ΔmtDNA molecules by long PCR, conventional PCR was used to more finely characterize deletion size differences on agarose gels (fig. 4) and for DNA sequencing analysis. The smaller amplicons generated using this approach allowed us to better determine the first experimental generation time point at which a particular variant could be detected and evaluate their ΔmtDNA sequence characteristics. In all the cases reported here, the follow-up secondary PCRs verified the deletion product predicted by the initial (and independently performed) long PCR. In most cases (14/19), once evidence of a new ΔmtDNA was observed on agarose gels (generation at first detection ranging from G0 to G45) it remained detectable through the end of the experiment at generation 50 (fig. 5). In five lines, the newly observed ΔmtDNA did not persist until G50. In three of these cases, a different new ΔmtDNA was detected in the same line within 5 or 10 generations and persisted through G50 (as in the case displayed by fig. 4). In the other two cases, evidence of the elements (ΔmtDNA-5, ΔmtDNA-8) disappeared and was replaced by ΔmtDNA-C gel bands in subsequent generations. These five exceptions occurred exclusively in two strain backgrounds (HK104, HK105) and at two population sizes (*N* = 1 and 10).

With only one exception, all of the new ΔmtDNA types were found in the *nad5* region of the mitochondrial genome where ΔmtDNA-C was originally discovered (table 2 and fig. 3). The single exception involved a deletion detected on the opposite side of the mtDNA chromosome, affecting *cytb*, *trnL*, *trnT*, and *nad4* gene sequences. This case was also unusual in that it was the sole incidence of a ΔmtDNA detected at *N* = 100, detection beginning at generation 30. Two of the new ΔmtDNA molecules had deletions that begin in ψnad5-2 and end in *nad5* (like ΔmtDNA-C) while the boundaries of six newly discovered deletions occurred entirely within the *nad5* gene. All of these deletions likely disrupt *nad5* protein-coding function. Like ΔmtDNA-C, all but one new deletion were flanked by directly repeated DNA sequence (table 2). However, with the exception of ΔmtDNA-6, the repeated flanking sequences are generally much shorter, at 7–9 bp, than that flanking the canonical deletion (21 bp).

The six *C. briggsae* progenitor strains displayed varying patterns of ΔmtDNA occurrence and evolution. In addition to ΔmtDNA-C which was observed in all progenitor strains, five new ΔmtDNA types were discovered in strain HK104, four in HK105, two in AF16, two in VT847, one in PB800, and zero in EG4181 (fig. 5 and table 2). We note that due to the extinction of most HK105 lines at *N* = 1, it is possible that additional undetected ΔmtDNA accumulated in these lines in their final generations. Some ΔmtDNA sequence types were observed in more than one *C. briggsae* strain background (table 2). ΔmtDNA-5 was detected in eight different experimental lines, across five progenitor strain backgrounds.

### Targeted Analysis of ΔmtDNA-C

In addition to our mitogenome-wide screen for new ΔmtDNA types, we also specifically targeted ΔmtDNA-C molecules using the PCR approach described in Howe and Denver (2008) and further implemented in Clark et al. (2012). Briefly, the PCR assay for levels of ΔmtDNA-C relative to intact mtDNA produces one of three banding patterns on agarose gels: a single large amplicon representing an intact mtDNA banding type, a single small amplicon representing the deletion banding type, or both bands indicating an intermediate type (Clark et al. 2012). Previous comparative analyses with quantitative real-time PCR (qPCR) data indicated that the standard PCR assays accurately represent distinctive bins with average proportions of ΔmtDNA-C being 0.04 (±0.06) for the intact band type, 0.30 (±0.15) for the intermediate band, and 0.60 (±0.07) for the deletion band (Howe and Denver 2008). We chose to use this rapid and inexpensive (though coarse) standard PCR method to track changes in the average heteroplasmic frequency of ΔmtDNA-C in the experimental populations studied here.

The vast majority of experimental lines (115/120) began with the intermediate-category ΔmtDNA-C gel banding pattern at G0. Likewise, the majority (86.4%) of the 1,271 total DNA samples analyzed (across all generations, genotypes, and population sizes) produced the intermediate banding type (fig. 5). The overall proportions of the intact and deletion band types were 5.6% and 8.0%, respectively. The deletion band type was not detected on any gels analyzing mtDNA from EG4181 and PB800, the two strains containing putative compensatory mutations (fig. 1*A*). The proportion of intact banding type among lines increased across generations in the *N* = 1,000 populations derived from strains EG4181 and PB800 (fig. 5). For example, in EG4181, 6/25 samples analyzed from G0 to G20 interval were scored intact, whereas 12/25 samples were scored intact from G30 to G50. Similar transgenerational shifts, but toward the deletion band type, were detected in population sizes of *N* = 1 and *N* = 10 from lines derived from four strains (AF16, VT847, HK104, HK105).

### Statistical Characterization of ΔmtDNA Evolution

The PCR approaches implemented in this study revealed the presence of previously known (ΔmtDNA-C) and novel ΔmtDNA variants. Although this approach offered a reproducible and sensitive method for identifying and characterizing new variants, the variably biased nature of PCR (smaller products generally amplify more efficiently than larger products) precluded efforts to quantify relative abundances of the many different ΔmtDNA circle types discovered across the greater than 1,000 nematode samples analyzed here. Thus, we based our analysis on the categorical gel banding patterns observed across generations and selected an ordered logistic regression statistical model where the outcome variable—deletion score—is ordered, but the quantitative distances between deletion categories are not assumed to be equal.

Five categorical deletion scores (1–5) were possible in our statistical analysis, with increasing scores reflecting increasing severity of ΔmtDNA state (see Materials and Methods for details). Explanatory variables examined in the statistical analysis were population size, strain, and generation number. A random effect was included for line to account for correlation of observations across generations for a given experimental line.

Likelihood ratio tests indicated that population size, strain, and generation number are significant predictors of ΔmtDNA score; the effect of generation number depended on population size (supplementary table S2, Supplementary Material online). Positive coefficients for population sizes of 1 and 10 indicated that evolution in small populations is strongly associated with increased ΔmtDNA prevalence (*P* = 0.004 and 0.001, respectively, table 3). At small population sizes (*N* = 1 and 10), ΔmtDNA scores were also more likely to increase with each generation, reflected by significant interaction effects between population size and generation number. However, in large populations, ΔmtDNA scores decreased over time, indicated by the negative coefficient of generation number. Note that ΔmtDNA scores were not significantly different in population sizes of 100 and 1,000, after accounting for other factors (*P* = 0.21).

ΔmtDNA scores were strongly associated with strain, with VT847, AF16, and HK104 having significantly greater odds of higher ΔmtDNA scores compared with EG4181 (all *P* < 0.001; *P* = 0.27 for PB800). The estimates in table 3 were exponentiated to obtain the odds of receiving a higher score relative to the reference category. Relative to a population size of 1,000, a population size of 1 was 22 times more likely to have a higher ΔmtDNA score (95% CI: 2.7–178). Relative to strain EG4181, strain AF16 was 232 times more likely to have a higher ΔmtDNA score (95% CI: 31–1,712).

**Fig. 6.**
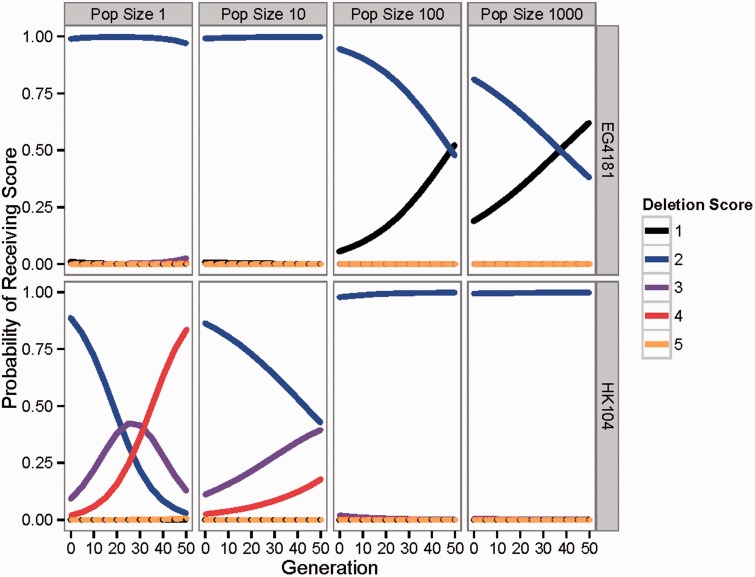
— Statistical predicted probabilities for ΔmtDNA categories. The probabilities of receiving ΔmtDNA categorical scores of 1–5 for strains EG4181 (top panels) or HK104 (bottom panels) at each population size studied. A score of 1 indicated the intact banding pattern (no ΔmtDNA of any type detectable, light blue squares in fig. 5), a 2 showed the intermediate banding pattern (both intact and ΔmtDNA-C, dark blue squares in fig. 5), and a 3 indicated the deletion pattern (ΔmtDNA-C band only visible on gel, dark red squares in fig. 5). A score of 4 showed the presence of a new ΔmtDNA type (bright red squares in fig. 5) and a 5 indicated the occurrence of second new ΔmtDNA type (arising after an initial new type scored as 4 in the same line, orange squares in fig. 5). Probabilities were calculated based on the ordered logistic regression model presented in table 3.

The overall statistical trends were illustrated by the probabilities predicted for each ΔmtDNA score based on the chosen model for a given generation number, strain, and population size (fig. 6). For instance, EG4181 had a very low likelihood of scoring 3–5 (categories of high ΔmtDNA prevalence) at any population size, and the likelihood of receiving a score of 2 or greater decreased for large population sizes (*N* = 100 and 1,000) as generation number increased. By contrast, HK104 was overall much more likely to receive band scores of 2 or greater as compared with EG4181; for population sizes of 1 or 10, the probability of a receiving a score above 2 increased dramatically as generation increased.

## Discussion

### Effect of Nematode Population Size

Our findings demonstrate that ΔmtDNA accumulates and diversifies in some experimental populations of *C. briggsae*, but only when population size is small (figs. 5 and 6). ΔmtDNAs are expected to confer a selective disadvantage due to negative consequences on organismal fitness through their adverse effects on mitochondrial functioning (Greaves and Turnbull 2009; Estes et al. 2011). At the between-molecule (mtDNA) level, however, ΔmtDNAs can experience directional selection associated with their transmission advantage relative to larger wild-type molecules (Diaz et al. 2002; Clark et al. 2012). When organismal population sizes are large, evolutionary theory (Otto and Hastings 1998; Rispe and Moran 2000) predicts that selective forces operating at lower (e.g., mtDNA) levels will be offset by selection among organisms evolving in populations. Consistent with this basic prediction, our study showed that ΔmtDNAs can persist and diversify at small nematode population sizes where drift is strong but selection is weak (e.g., *N* = 1 in HK104), but can be subject to selective elimination in larger nematode populations (e.g., *N* = 1,000 in EG4181)—see figures 5 and 6 and table 3. At *N* = 1 we observed that it was possible for new ΔmtDNAs of smaller genome size to arise and quickly outcompete previous ΔmtDNAs of larger size across generations (fig. 4). This observation shows that, when population size is small, ΔmtDNA is capable of not only expanding but also diversifying into new forms.

We observed that preexisting ΔmtDNA levels sometimes decreased across generations, but only when nematode population size was large (*N* = 1,000). In PB800, for example, levels of ΔmtDNA-C were observed to be intermediate (sensu the Howe and Denver 2008 PCR assay) at generations 0 and 5 in all *N* = 1,000 lineages (figs. 5 and 6). In later generations, however, intact ΔmtDNA-C band scores were much more prevalent, indicating a trend of ΔmtDNA-C levels decreasing across generations. This observation suggests that ΔmtDNA-C molecules in PB800 were subject to selective elimination, but only when population size was large (*N* = 1,000). A similar pattern was observed for HK104. At generations 0, 5, and 10, the deletion ΔmtDNA-C band category was prevalent in the *N* = 1,000 experimental lines. At generation 15 and beyond, however, the intermediate category predominated, again indicating that ΔmtDNA-C levels decreased across generations. For the other three *C. briggsae* strains (AF16, HK104, VT847), more static patterns were observed across generations at *N* = 1,000. This observation is particularly noteworthy for strain AF16 where high male frequencies were observed in the *N* = 100 and *N* = 1,000 lines (supplementary table S1, Supplementary Material online). The similarity between AF16 and other strains lacking compensatory alleles, rather than EG4181 and PB800, suggests that the prevalence of males had a minimal impact on ΔmtDNA-C evolution in AF16. Our combined results implicate host population size as a key factor that shapes the evolutionary trajectories of naturally occurring ΔmtDNA elements.

ΔmtDNA variants must traverse a complicated biological path to being a stable variant in a natural animal population, as is observed in *C. briggsae.* All such elements are initially born from a de novo deletion mutation and into immediate coexistence with larger intact mtDNA molecules inside the same mitochondrion. Little is known, however, about the spread and distribution of such elements across levels of biological complexity (fig. 2). At the cellular and single-organism levels, mitophagy and mitochondrial fission/fusion pathways play critical roles in mitochondrial quality control (Mishra and Chan 2014). It is possible that ΔmtDNAs might take advantage of mitochondrial fission/fusion cycles as a mechanism for spreading throughout a eukaryotic cell, though experiments remain to be done to test that hypothesis. At the population level, ΔmtDNA-C levels are expected to be controlled by a combination of purifying selection and a transmission advantage, which together could be permissive to the spread of ΔmtDNA when populations are small and purifying selection is weak. We lack basic knowledge on the distribution of ΔmtDNA molecules across mitochondria and cells within an individual nematode which limits our ability to understand the evolutionary dynamics and fitness impacts of these elements.

### Effect of Nematode Strain

Although population size was a major factor in determining patterns of variation in ΔmtDNA accumulation in our study, we also observed variable patterns of accumulation among nematode strains. For example, in HK104, new ΔmtDNA molecules arose and persisted at *N* = 1 and, to a lesser extent, at *N* = 10, with no new ΔmtDNA types and mostly static ΔmtDNA-C patterns observed across generations at *N* = 100 and *N* = 1,000 (figs. 5 and 6). Similarly dynamic patterns were observed at small population sizes (*N* = 1 and 10) in HK105, and to a lesser extent in VT847, the strains previously determined to harbor the highest natural levels of ΔmtDNA-C (Howe and Denver 2008). By contrast, no new ΔmtDNA molecules were found to arise in EG4181 at smaller population sizes (*N* = 1, 10, and 100); instead, static ΔmtDNA-C patterns were observed. As previously discussed, EG4181 harbors putative compensatory mtDNA mutations that are associated with lower overall ΔmtDNA-C levels as compared with strains lacking those mutations. The compensatory mutations also occur in PB800 where similar static patterns were observed in smaller populations. The differential occurrence of compensatory mtDNA mutations among strains might explain the relative stability of ΔmtDNA-C levels in EG4181 and PB800 compared with the other four strains. However, this factor cannot explain the occurrence of new ΔmtDNA types (involving different direct repeats compared with those associated with ΔmtDNA-C) in AF16, HK104, HK105, and VT847 strains but not in EG4181 and PB800. We searched for potential compensatory mutations in the direct repeats associated with new ΔmtDNAs discovered here but discovered none in EG4181, PB800, or the other four strains. This suggests that the low occurrence of all ΔmtDNA types in EG4181 and PB800 might result from superior mitochondrial quality control (e.g., via mitophagy pathways) and/or lower baseline mtDNA deletion formation rates as compared with the other four strains. These observations also suggest that ΔmtDNA might be more deleterious in EG4181 and PB800 genetic backgrounds as compared with the other four strains.

The AF16 progenitor strain evolved higher male frequency in its *N* = 100 and 1,000 lines compared with those of the other five progenitor strains (*N* = 1,000 results summarized in supplementary table S1, Supplementary Material online). Why elevated male frequency was observed for AF16 alone remains a mystery, but the fact may have impacted our observations in at least two ways. First, if the ΔmtDNA elements studied here have any direct selective effects on males (unknown) or if their transmission was biased toward male offspring, ΔmtDNA elements in these lines would have been exposed to an additional form of selection compared with those in all other lines where males were essentially absent (Beekman et al. 2014). Second, to the extent that increased male frequency led to successful outcrossing in *N* = 100 and 1,000 AF16 lines, we would expect the *N_e_* of nuclear genes in these replicate lines to be increased relative to those of the other strains. This, in turn, might increase the efficiency of selection against ΔmtDNA elements through nuclear suppressor loci in the larger, male-rich AF16 lines than in lines of other strains experiencing identical bottleneck (transfer) sizes. If true, increased selection efficiency afforded by sexual reproduction may have helped to limit ΔmtDNA accumulation—or in some cases to eliminate ΔmtDNA elements—in this strain (fig. 5).

### ΔmtDNA Origins and Heteroplasmic Segregation Patterns

Ten different ΔmtDNA types were detected in the present study (table 2). The ΔmtDNA-C molecule was the most predominantly observed ΔmtDNA type in this study, as expected due to its known occurrence in natural *C. briggsae* populations and progenitor strains used here. For 7/9 new *C. briggsae* ΔmtDNA types reported here, the elements were discovered only once in a single experimental nematode lineage; all of these cases likely represent de novo deletion mutation events that occurred during the experiment. Two ΔmtDNA types (ΔmtDNA-5, ΔmtDNA-6), however, were found in more than one experimental line, and also from two or more different progenitor strains.

ΔmtDNA-5 was detected in seven different experimental lines (fig. 5 and table 2). This element was detected in 5/30 *N* = 1 lines and 2/30 *N* = 10 lines. ΔmtDNA-5 was found in 5/6 progenitor strain backgrounds (all except EG4181). Is it possible that these patterns might be entirely explained by de novo deletion mutation formation (rather than segregation of preexisting heteroplasmic variants) during the experiment? A mutation rate of 3.3 × 10^−^^3^ would be required for de novo mutation to explain the observed occurrences of ΔmtDNA-5 at *N* = 1 in our study. This value is approximately 3 times higher than the rate for new deletion formation in *C. briggsae* mtDNA reported in Howe et al. (2010). The possibility that this locus represents a region that is hypermutable for deletion formation cannot be ruled out. However, in HK104 this specific element was observed at the earliest timepoint assayed (G5) two times. In AF16, detection of ΔmtDNA-5 first occurred once at G5 and at G15 in another line. In one HK105 lineage, the element was first detected at G10. These observations suggest that, at least in some cases, ΔmtDNA-5 already existed in the progenitor nematode strains at the onset of the experiment and underwent differential segregation in different lines.

ΔmtDNA-6 was found to arise and persist in one *N* = 10 lineage of HK104, and one *N* = 10 lineage of VT847. In both cases, the first evidence of ΔmtDNA-6 occurred midway through the experiment (G25 in HK104 and G30 in VT847). The observation of this element at *N* = 10 only is peculiar when compared with patterns at other ΔmtDNA elements which predominated at *N* = 1. These observations suggest that ΔmtDNA-6 probably arose through de novo mutation and accumulated to detectable levels two independent times during the experiment. Our combined observations suggest that *C. briggsae* ΔmtDNA elements likely evolve through a complex combination of high baseline mutation rates of de novo formation (Howe et al. 2010), poorly understood segregation across mitochondria and cells, and purifying selection at the level of nematode populations.

### Implications for Evolution and the Study of *C. briggsae*

More than a decade ago, an experimental evolution study of yeast petite mutants yielded landmark basic insights into the role of population size in governing the evolution of selfish mtDNA (Taylor et al. 2002). Our main finding that *C. briggsae* ΔmtDNA is also greatly influenced by host population size is consistent with the earlier yeast study, but also provides new insights at the molecular-genetic level and into the role of host genetic background (figs 5 and 6). This work also builds substantially on recent nematode experimental evolution work performed by ourselves and others (Howe et al. 2010; Gray and Cutter 2014), providing a study with five-generation-level resolution and four different experimental population sizes considered. Despite these advances many basic mysteries about ΔmtDNA evolutionary trajectories in *C. briggsae* remain, such as its segregation across cells within an individual nematode. We also lack knowledge on its potential impact on males where evolutionary theory predicts ΔmtDNA might have disproportionately deleterious effects (Aanen et al. 2014).

Why are such heritable ΔmtDNA molecules found in *C. briggsae* but not more broadly throughout animal phylogeny? It is possible that long-term evolution in small *N_e_*, exacerbated by the androdioecious reproductive mode of this species, might render natural selection at the population level too weak to effectively combat ΔmtDNA accumulation in *C. briggsae.* Our study demonstrated that evolution at *N* = 1,000 is often insufficient to purge ΔmtDNA across 50 generations (fig. 5). Thus, natural *C. briggsae* populations evolving in such population-genetic environments might be sufficient to explain the long-term persistence of ΔmtDNA in this species. *Caenorhabditis briggsae* is also known to harbor another, presumably X-linked, selfish gene system that results in sex ratio distortion (hermaphrodite overabundance) in male sperm-fertilized progeny (LaMunyon and Ward 1997). Natural populations of *Caenorhabditis elegans*, a congener of *C. briggsae* that independently evolved androdioecious reproduction (Kiontke et al. 2004), are not known to harbor ΔmtDNA in nature but do contain a nuclear two-gene (*zeel-1*/*peel-1*) selfish DNA system (Seidel et al. 2011). Although *C. briggsae* might be unusually prone to selfish element accumulation, it is possible that ΔmtDNA elements are more widespread across many different forms of metazoan life, but remain cryptic owing to the expected conservative tendencies of investigators to dismiss evidence of their occurrence (e.g., smaller-than-expected PCR amplicons detected alongside larger target amplicons) as technical error.

Our study demonstrated that the evolutionary trajectories of naturally occurring ΔmtDNA molecules follow the basic rules of population-genetic theory: that they proliferate when host (organism) population size is small, and decline when host population size is large. It was also revealed that different natural strains (i.e., genetic architectures) of *C. briggsae* are differentially susceptible to the accumulation of deleterious ΔmtDNA. Knowledge on the (presumably) nuclear genome-encoded determinants of this variation will require future studies involving recombinant inbred line approaches (Ross et al. 2011). We also make an important practical note here that the high degree of ΔmtDNA plasticity across generations has potentially large implications for researchers studying *C. briggsae*—bottlenecking nematodes might lead to rapid changes in ΔmtDNA levels and have an impact on phenotypes under study. This might also contribute to some differences in strain-specific life history traits measured for the same *C. briggsae* strain, but by different research groups (e.g., HK104 fecundity values reported in Estes et al. (2011) was much lower than values reported in Dolgin et al. (2007). Future studies are needed to understand the broader relevance of ΔmtDNA to *C. briggsae* in its natural environment, the within-organism and within-cell distribution patterns of ΔmtDNA, and its possible roles in mating system evolution, speciation, and genetic incompatibility.

## Supplementary Material

Supplementary figure S1 and tables S1 and S2 are available at *Genome Biology and Evolution* online (http://www.gbe.oxfordjournals.org/).

Supplementary Data
