# Supplementary material for: Selfish Mitochondrial DNA Proliferates and Diversifies in Small, but not Large, Experimental Populations of Caenorhabditis briggsae
Source: Genome Biol Evol. 2015 Jun 24;7(7):2023–37. doi: 10.1093/gbe/evv116 (PMC4524483; doi:10.1093/gbe/evv116)
Supplement: Supplementary Data [file supp_7_7_2023__index.html]

Selfish Mitochondrial DNA Proliferates and Diversifies in Small, but not Large, Experimental Populations of Caenorhabditis briggsae — Supplementary Data 

# Selfish Mitochondrial DNA Proliferates and Diversifies in Small, but not Large, Experimental Populations of *Caenorhabditis briggsae*

## Supplementary Data

files

- Supplementary Data - pdf file
