# Supplementary material for: Selfish Mitochondrial DNA Proliferates and Diversifies in Small, but not Large, Experimental Populations of Caenorhabditis briggsae
Source: Genome Biol Evol. 2015 Jun 24;7(7):2023–37. doi: 10.1093/gbe/evv116 (PMC4524483; doi:10.1093/gbe/evv116)
Supplement: Supplementary Data [file supp_evv116_Phillips_GBE_Revision_SuppInfo.pdf]

Fig. S1. Site-by-site mtDNA coverage in high-throughput DNA sequencing data. Data derived from Illumina MiSeq DNA sequencing for DNA from L1-staged unevolved AF16 nematodes (black line) and L1-staged AF16-derived nematodes that were bottlenecked for 50 generations (orange line). The two samples were run in multiplex at equal molar ratios in a shared MiSeq run. Raw site-by-site coverage values are shown. The distinctive dip in coverage indicated by the arrow in the evolved line is in *nad5* and nearby regions where ΔmtDNA-C is known to occur. See Materials and Methods for more details.

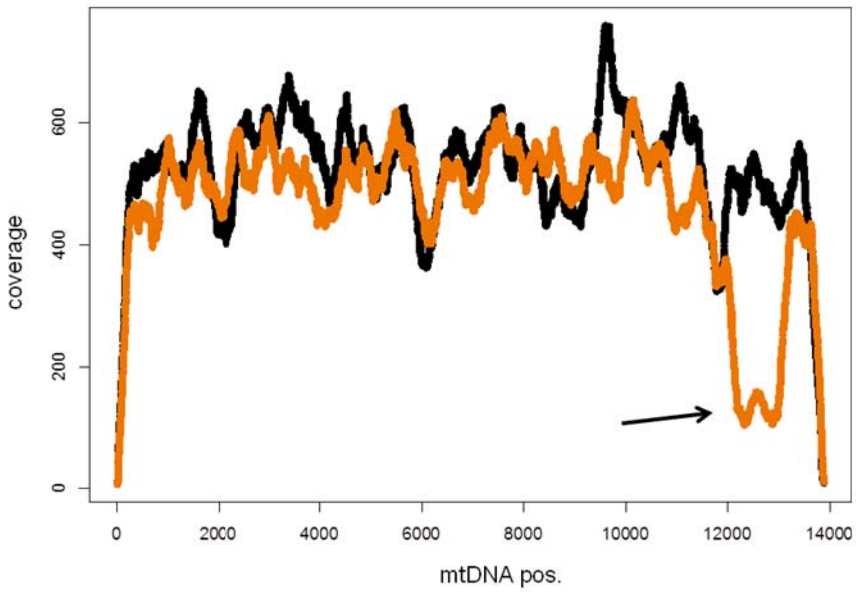

Table S1. *C. briggsae* male frequencies in *N*=1000 treatment. Male frequencies were estimated at a single generational time point (G41) from 150 individuals from each of the five replicates per strain.

**Strain**	**Avg. Male Freq.**	**St. Dev.**
AF16	0.2206	0.1171
EG4181	0.0022	0.0049
HK104	0.0044	0.0010
HK105	0.0000	0.0000
PB800	0.0038	0.0055
VT847	0.0027	0.0037

Table S2. Model selection results for ordinal logistic regression analysis. Likelihood ratio tests were used to find the most parsimonious model by singly dropping each explanatory variable (models on lines 2-4 in table compared to full model on line 1). After determining that all 3 explanatory variables should be included in the model (p-value less than 0.05 indicating that the extra variability explained by the full model is statistically significant), we tested for significance of all possible pairwise interactions among the variables (models on lines 5-7 compared to reduced model on line 1). Likelihood ratio (LR) statistics are based on a chi-square distribution comparing each subsequent model to the model on the first line. The selected model, with the lowest Akaike Information Criterion (AIC) and highest log-likelihood, was Strain + Gen x Pop size.

Explanatory variables	Number parameters	AIC	Log-likelihood	*LR*	*LR d.f.*	P-value
Strain + Gen + Pop size	13	887.27	-430.63	NA	NA	NA
Strain + Gen	10	947.92	-463.96	66.65	3	<0.001
Strain + Pop size	12	890.42	-433.21	5.16	1	0.023

Gen + Pop size	9	936.30	-459.15	57.03	4	<0.001
Strain + Gen x Pop size	16	819.43	-393.72	73.83	3	<0.001
Strain x Gen + Pop size	17	846.17	-406.08	49.10	4	<0.001
Strain x Pop size + Gen	25	879.91	-414.95	31.36	12	0.0017
